# A Procalcitonin and C-Reactive Protein-Guided Clinical Pathway for Reducing Antibiotic Use in Children Hospitalized with Bronchiolitis

**DOI:** 10.3390/children8050351

**Published:** 2021-04-28

**Authors:** Elisa Barbieri, Sara Rossin, Carlo Giaquinto, Liviana Da Dalt, Daniele Dona’

**Affiliations:** 1 Division of Paediatric Infectious Diseases, Department of Women’s and Children’s Health, University of Padova, 35100 Padova, Italy; carlo.giaquinto@unipd.it (C.G.); daniele.dona@unipd.it (D.D.); 2 Pediatric Emergency Department, Department of Women’s and Children’s Health, University Hospital of Padova, 35100 Padova, Italy; sara.rossin@gmail.com (S.R.); liviana.dadalt@unipd.it (L.D.D.)

**Keywords:** procalcitonin, bronchiolitis, paediatric emergency department, clinical pathways

## Abstract

Despite the lack of evidence that bronchodilators, corticosteroids, and antibiotics are useful in treating bronchiolitis, their use is still widespread. This study aimed to determine the consumption of antibiotics for bronchiolitis before and after a procalcitonin-guided clinical pathway (CP) implementation. In December 2019, a CP for lower respiratory tract infection management was implemented at the Department of Women’s and Children’s Health at Padua University Hospital. This was a pre-post, quasi-experimental study that assessed the changes in the treatment of bronchiolitis during two bimesters preceding the CP implementation (pre-period: January 2018–February 2018 and January 2019–February 2019) and during the bimester after CP implementation (post-period January 2020–February 2020). After the CP implementation, there was a significant reduction in antibiotic prescriptions from 36.2% to 12.5% (*p* = 0.036) in patients hospitalized for bronchiolitis. Co-amoxiclav treatment, the antibiotic most commonly administered, decreased from 66.6% to 33.3%. Among outpatients’ bronchiolitis episodes, a statistically significant decrease in beta2-agonists’ use (from 18.0% to 4.4%, pre and post periods) and a quasi-significant decrease in corticosteroid use (from 8.0% to 0% pre and post periods) were observed. An evidence-based CP supported by educational lectures was associated with significant changes in the physicians’ prescribing habits.

## 1. Introduction

Bronchiolitis is the most common lower respiratory tract infection (LRTI) in children less than one year of age and has a mainly viral etiology [1,2,3]. It is a self-limiting disease; therefore, management should focus on supportive care based on oxygen therapy and fluid supplementation [4]. Despite the lack of evidence that bronchodilators, corticosteroids, and antibiotics are useful in slowing the course of this disease and in treating bronchiolitis, their use is still widespread [5]. Moreover, the lack of clinical, radiological, and laboratory tests to safely rule out bacterial involvement in LRTI still drives antibiotic treatment today [6]. It is mandatory to reduce over-prescription for bronchiolitis, especially with antibiotics, because of increased resistance. 

In the presence of bacterial infection, the biomarker procalcitonin (PCT) is produced in the parenchymal tissues mediated by cytokines IL-6, TNF-α, and IL-β, and the degree of PCT rise correlates with the severity of the infection. Conversely, PCT production is attenuated by interferon-γ primarily secreted in response to viral infection [6,7,8,9,10].

The success of PCT guidance in adults with LRTI also triggered pediatric research and proved to be effective in reducing antibiotic exposures in primary care and hospital settings [11,12,13]. Despite the implementation, there is still no consensus on the cut-offs to be considered for children [14,15,16,17].

The primary aim of this study was to determine the consumption of antibiotics for bronchiolitis before and after the implementation of a PCT/PCR-guided clinical pathway (CP). As a secondary aim, we determined the prescription rates of inhaled beta-agonist, corticosteroids, and epinephrine before and after the CP implementation.

## 2. Materials and Methods

### 2.1. Study Design

This was a quasi-experimental study that assessed the changes in antibiotic prescribing for bronchiolitis during two bimesters before (pre-period: 1 January 2018, 28 February, 2018; 1 January 2019, 28 February 2019) and one bimester after CP implementation (post-period: 1 January 2020, 28 February 2020). The study was conducted at the Pediatric Emergency Department (PED) and Pediatric Acute Care Unit (PACU) of the Department of Women’s and Children’s Health (DWCH) of Padua University Hospital. This hospital provides primary and secondary care for a metropolitan area of 350,000 people and tertiary care for a regional and extra-regional population, with approximately 24,000 PED visits per year and an overall hospital admission rate from PED of around 7%.

### 2.2. Intervention and Clinical Pathways

In December 2019, the CP for the management of LRTI was implemented and presented to physicians and residents working in the PED and PACU (Appendix A—Figure A1).

The CP is a one-page decision support algorithm designed to assist providers in determining whether antibiotic treatment should be started or not. This PCT/PCR-guided algorithm summarizes international guidelines and was developed by a multidisciplinary group (pediatric infectious diseases specialist and microbiologist) at the DWCH at Padua University Hospital, where it is in place for more than four years an efficient antibiotic stewardship program based on CPs.

Although bronchiolitis diagnosis is clinical, and blood tests and chest X-ray exams are not routinely necessary, for children hospitalized with severe bronchiolitis with a suspicion of co-infection it is suggested to add PCT to the classical blood exams.

CP indicates that with: (i) PCT < 0.25 μg/L in addiction to C-reactive protein (CRP) < 100 mg/L—a bacterial infection is unlikely and the initiation or continuation of antibiotic is strongly discouraged; (ii) PCT > 0.25 μg/L and CRP >100 mg/L—a possible bacterial infection and the initiation or continuation of an antibiotic therapy is encouraged; (iii) PCT > 0.25 μg/L and PCR < 100 mg/L or PCT < 0.25 μg/L and PCR > 100 mg/L—the decision to start or continue the antibiotic therapy should be based on clinical examination.

Antibiotic therapy can be considered in critically ill patients (i.e., unstable vital signs or ICU admission). If antibiotics are given, early discontinuation after 1, 3, or 5 days should be endorsed if PCT levels remain < 0.1 μg/L.

### 2.3. Study Population and Exclusion Criteria

All patients aged between 1 and 12 months with an International Classification of Diseases, 9th Revision, Clinical Modification code, or descriptive diagnosis of bronchiolitis were included.

Patients with a concomitant bacterial infection, ongoing antibiotic therapy (i.e., defined as an antibiotic prescription in the 30 days before the bronchiolitis case), immunodeficiency or immunosuppressive therapy, chronic disease (including cystic fibrosis and diabetes), sickle cell anemia, Down’s syndrome or congenital cardiac disease, or chronic lung disease (except asthma) were excluded.

Admissions for bronchiolitis occurring in the same patient greater than 30 days apart were analyzed as separate events.

### 2.4. Data Collection

All clinical, demographic, diagnostic, and prescription data were retrieved from Galileo^®^ and manually collected from electronic medical records through the Redcap^®^ data collection form.

The following information was considered: date of birth, admission and discharge dates, the reason for discharge, descriptive diagnosis, sex, body weight, a previous visit to the primary care pediatrician (i.e., within seven days), antibiotic allergy, and vaccinations. Illness severity was assessed as mild, moderate, or severe by SR and DD according to respiratory rate, respiratory effort, oxygen saturation, feeding rate, and apnea. Other data required were diagnostic exams (nasopharyngeal swab, chest X-ray, complete blood cell count, CRP, and PCT) and treatments (antibiotics, inhaled glucocorticoids, inhaled beta2-agonists, and inhaled epinephrine).

Privacy was guaranteed by assigning each patient a unique study-specific number and not collecting personally identifying data.

The investigations were carried out following the rules of the Declaration of Helsinki of 1975 (https://www.wma.net/what-we-do/medical-ethics/declaration-of-helsinki/ accessed on 14 January 2021), revised in 2013. This study was approved by the Ethical Committees of Padova University Hospital (3737/AO/16). Due to the nature of the study, (observational retrospective) no informed consent was required.

### 2.5. Outcomes

The primary outcome measured was the number of episodes receiving antibiotic treatment by type of antibiotic compared to the number of total episodes in the various periods considered.

The explorative outcomes were the number of episodes receiving inhaled beta-agonists, the number of episodes receiving corticosteroids, and the number of episodes receiving epinephrine.

For inpatients, medicine administered within four days from the admission date was considered related to the event.

### 2.6. Data Analysis

Results were summarized as frequencies and percentages (categorical variables) and as means with standard deviations (SD) or medians with interquartile ranges (IQRs) (continuous variables) for both inpatients and outpatients. Comparison of categorical variables in the pre vs. post-periods was conducted with χ2 or Fisher exact test. Continuous variables were compared with the non-parametric Mann–Whitney U test or one-way ANOVA. A *p* value of < 0.05 was considered to be statistically significant for all statistical tests. Data were analyzed using R statistical software (version 3.6.3, Vienna, Austria).

## 3. Results

Overall, 82 inpatients (58 in the pre-period and 24 in the post-period) and 145 outpatients (100 in the pre-period and 45 in the post-period) were included. Three children were excluded due to the presence of concomitant disease and because of ongoing antibiotic therapy.

### 3.1. Inpatients

Demographic characteristics, signs and symptoms, and microbiological and laboratory-related data are shown in Table 1.

No difference was found in sex prevalence nor age, but in the pre-period cohort, in 15% of cases, children were born < 37 weeks of age. Not surprisingly, most of the patients were not vaccinated (72.4% and 45.8% in the pre- and post-periods). Patients had a previous visit with their primary care pediatricians in 39.7% and 35.5% of inpatient episodes in the pre-period and post-period, respectively; in 39.1% (pre-period) and 33.3% (post-period) of cases, the primary care pediatricians requested an ER visit (Table 1).

The virus most frequently found in the nasopharyngeal swab analysis was RSV (8.9% and 20.8% in the pre- and post-periods), followed by rhinovirus and parainfluenza virus. Clinical analysis requests and results were similar in the two cohorts, except for PCT requests, which increased significantly in the post-period (29.3% vs. 70.8%, pre-period vs. post-period, *p* = 0.001). Median PCT values did not change in the periods considered (Table 1).

Antibiotic prescription after CP implementation decreased significantly from 36.2% to 12.5% (*p* = 0.036). Co-amoxiclav alone was the most prescribed antibiotic in the pre-period (76.2%), followed by ceftriaxone and the combination of ceftriaxone with a subsequent shift to co-amoxiclav (9.5% each) and azithromycin (4.7%). The three antibiotic therapies administered in the post-period were: co-amoxiclav, ceftriaxone/co-amoxiclav, and ceftriaxone/ampicillin (Table 2).

Betamethasone was administered just in two cases in the pre-period. Inhaled beta2-agonist (salbutamol) was administered in 6.9% and 20.8% of cases in the pre- and post-periods, respectively. Inhaled epinephrine was administered just in three cases in the pre-period (Table 2).

### 3.2. Outpatients

The cohorts in the pre- and post-periods were similar with respect to sex and age (Table 1). Around 30% of outpatients were not vaccinated, and overall in 47.5% of cases, children were previously visited by a primary care pediatrician who requested an ER visit—38.7% and 25.0% of cases in the pre- and post-periods, respectively. (Table 1)

The oxygen saturation median rate was above 98% in both pre- and post-periods, and an increase in nasopharyngeal swab requests in the post-period was noted (*p* = 0.001), resulting in four swabs positive for rhinovirus and four swabs positive for RSV. Other diagnostic test requests and results were similar in both periods, except for PCT requests, which increased in the post-period, with no changes in median value results. (Table 1)

Only one patient in the pre-period received antibiotic treatment, and amoxicillin was administered. Inhaled glucocorticoids were prescribed in the pre-period in 8% of cases with a quasi-significant decrease (*p* = 0.058). Inhaled beta2-agonist prescription significantly decreased in the post-period (from 18% to 4.4%, pre- and post-periods, respectively; *p* = 0.036; Table 2).

## 4. Discussion

Our results indicate that the CP’s implementation was significant in decreasing antibiotic prescription rates in patients hospitalized with bronchiolitis. No variation was reported in the outpatient group.

In line with the literature, children’s median age was lower for inpatients with respect to outpatients, and in both populations, the two cohorts in pre- and post-periods were similar with respect to sex and age.

After the implementation of this CP, there was a significant increase in PCT measurements not only among inpatients but also among outpatients. It should be noted that bronchiolitis is a clinical diagnosis, and blood tests and chest X-rays, are not routinely recommended in line with national and international guidelines [1,18,19]. However, other studies aiming to reduce antibiotic prescription in infants with severe bronchiolitis have investigated the use of PCT and PCR biomarkers [20,21].

Following this, our study suggested that clinicians recognize the validity of this marker in supporting the differential diagnosis in children hospitalized with severe bronchiolitis, for which there is a strong suspicion of a bacterial co-infection.

It has previously been reported in the literature that uncertainties around diagnosis and fear of bacterial co-infection are drivers of overprescribing of antibiotics and other non-evidence based prescription in children with bronchiolitis. Indeed, Carande and colleagues demonstrated that almost half (49%) of pediatricians included in a study set in a UK primary care setting were prescribed medication to treat potential differential diagnoses, reflecting the clinical challenge of differentiating between bronchiolitis and other conditions (i.e., pneumonia or recurrent wheezing) [22]. In our study, prescription of antibiotics (especially before CP implementation) and PCT testing (especially after CP implementation) might represent clinicians’ fear of co-infection with a resistant strain of *S. pneumoniae*, since co-amoxiclav was used instead of amoxicillin.

Another challenging fact that should be taken into account when discussing patient care in children with bronchiolitis is the vaccination status; indeed, in our study we found very few children had received complete immunization, including pneumococcal vaccine.

Among outpatients, a decrease in antibiotic prescriptions was not significant, but it was expected. In the PED of Padua University Hospital, an antimicrobial stewardship program was implemented, which resulted in a significant decrease in broad-spectrum prescription rates that might have an impact on clinicians’ behaviors toward antibiotic prescribing for outpatients [23,24]. Moreover, it should be considered that patients hospitalized present more severe symptoms.

Even though decreasing inhaled beta2-agonists was not the primary aim of this study, a statistically significant decrease in salbutamol prescription rates for outpatients was noted after the CP implementation. The first part of the CP is designed to help clinicians assess a correct differential diagnosis that is subsequently categorized according to illness severity as mild, moderate, or severe. Beta2-agonists seem ineffective in the resolution of the disease, when used without evidence, as already reported in the Italian outpatient setting [25]. Thus, the CP might have also helped in the severity assessment.

Furthermore, a quasi-significant decrease of corticosteroids prescribed among outpatients was observed. Systemic and nebulized steroids did not decrease the incidence and duration of hospitalization or improve short and long-term prognosis.

Our study has strengths and limitations. It is one of the first studies evaluating the effectiveness of an Antimicrobial Stewardship Program (ASP) based on a CP in an Italian hospital. This intervention was designed to be feasible and was developed by a multidisciplinary team to guarantee a high quality and level of coordination, with cooperation between the infectious diseases and PED teams. To our knowledge, it was the first study in which a CP was used to lead pediatricians’ decisions to a correct diagnosis and treatment of children with bronchiolitis.

Limitations of this study include the quasi-experimental design, the fact that just one center was involved, and the short period of observation. Despite the randomized controlled trial representing a better study design in terms of reduced bias, it is also known that is costly and time-consuming; hence, we believe that implementing a stewardship policy or quality initiative could be a valid alternative. Indeed, our results were similar to a randomized control study conducted in Switzerland to assess PCT-guided treatment, which resulted in a decrease by 28 percentage points in antibiotic prescribing rate for non-CAP LRTI [26]. On the other hand, researchers assessed the preliminary hypothesis of PCT usefulness in children, and in fact, PCT cut-offs were derived from adult data and thus thought not to be the best option. Second, in the two months after CP implementation, a 70% decrease in emergency room visits was noted linked to the restrictions following the worldwide pandemic caused by SARS-CoV-2. Hence, to obtain better estimates on the CP’s impact and its sustainability over time, further data should be collected in the next seasons. Third, although PCT is more precise than CRP at identifying viral diagnosis, it is not a specific biomarker and there is no international agreement on PCT cuts-off in children [21,27,28]. In recent years, rapid tests constituted by a combination of biomarkers such as CRP and myxovirus resistance protein A (MxA) have proven to be very sensible and specific when differentiating between infections of bacterial and viral etiology. Unfortunately, data on children less than two years old, such as the bronchiolitis population, are scarce [29]. Fourth we included in the CP algorithm the possibility to conduct blood tests in particular cases, and this might have been misinterpreted by some clinicians as an excuse to conduct PCT blood testing in non-severe bronchiolitis episodes in outpatients. A possible solution could be represented by introducing a warning note in the algorithm reminding the clinicians that such exams are not routinely performed, as with chest X-rays. Fifth, the impact of the biomarker exams on costs might represent a limitation, especially if economic resources are scarce (in our center the cost of each PCT test was 14.40 EUR and that of each CRP test was 4.20 EUR).

Differently from guideline publications alone, [30] stewardship policy or quality improvement represent valid strategies to decrease inappropriate prescriptions mainly driven by challenges in defining the diagnosis etiology, over-expectations regarding treatments, and perceived parental pressure toward receiving antibiotic prescriptions for outpatients. Moreover, previous studies conducted in hospital settings have demonstrated the usefulness of implementing QI measures at a local level or with collaboration between different settings to improve the management of acute bronchiolitis [21,31].

The unnecessary use of antibiotics in patients with LRTI having mainly a viral cause, such as bronchiolitis, impacts patient care and the development of antibiotic resistance worldwide.

## 5. Conclusions

The evidence-based CP supported by educational lectures has proved to be an efficient strategy to disseminate best practices to reduce antibiotic use in hospitalized patients and via inhaled beta2-agonist prescriptions for outpatients, and the adherence to bronchiolitis guidelines increased. To avoid unnecessary use of diagnostic testing, particular emphasis should be placed in the development of the clinical pathway algorithm.

## Figures and Tables

**Table 1:** Demographics, signs and symptoms, and microbiological and diagnostic exams of patients with bronchiolitis. Statistical comparison was performed only for symptoms, and microbiological and diagnostic exams; and only significant *p* values are reported.

	Inpatients			Outpatients		
	Pre-Intervention Period	Post-Intervention Period	*p*-Value	Pre-intervention Period	Post-Intervention Period	*p*-Value
**N. of included episodes**	58	24		100	45	
**Sex, female (%)**	31 (53.4)	15 (62.5)		43 (43.0)	25 (55.6)	
**Age in months, Mean (SD)**	2.24 (1.80)	2.96 (2.42)		4.32 (2.87)	3.82 (2.27)	
**<37-week gestation, Yes (%)**	9 (15.5)	1 (4.2)		2 (2.0)	1 (2.2)	
**Bodyweight, kg, Mean (SD)**	5.13 (1.25)	5.51 (1.51)		6.93 (1.88) ^c^	6.57 (1.55)	
**Vaccination, (%)**			0.04			
No	42 (72.4)	11 (45.8)		31 (31.0)	13 (28.9)	
Unknown	5 (8.6)	2 (8.3)		10 (10.0)	1 (2.2)	
Yes	11 (19.0)	11 (45.8)		59 (59.0)	31 (68.9)	
Completed immunization plan	2 (18.2)	6 (54.5)		30 (50.8)	13 (28.9)	
**Previous visit by primary care pediatrician, Yes, (%)**	23 (39.7)	9 (37.5)		49 (49.0)	20 (44.4)	
ER visit request, Yes, (%)	9 (39.1)	3 (33.3)		19 (38.7)	5 (25.0)	
**Illness severity, (%)**						
Mild	9 (15.5)	1 (4.2)		94 (94.0)	42 (93.3)	
Moderate	48 (82.8)	17 (70.8)		6 (6.0)	3 (6.7)	
Severe	1 (1.7)	6 (25.0)		0 (0)	0 (0)	
**Symptoms, (%)**						
Coryza	15 (25.9)	9 (37.5)		20 (20.0)	22 (48.9)	
Cough	12 (20.7)	2 (8.3)		33 (33.0)	13 (28.9)	
Wheezing	14 (24.1)	6 (25.0)		30 (30.0)	13 (28.9)	
Crackles	47 (81.0)	19 (79.2)		81 (81.0)	33 (73.3)	
Tachypnea	39 (67.2)	20 (83.3)		44 (44.0)	21 (46.7)	
Chest retractions	36 (62.1)	14 (58.3)		31 (31.0)	9 (20.0)	
Body temperature ≥ 37.5 °C	9 (15.5)	5 (20.8)		22 (22.0)	7 (15.6)	
**O_2_** **Saturation, Mean (SD)**	95.5 (2.95)	95.1 (3.91)		98.3 (1.62) ^a^	98.7 (1.29) ^b^	
**Limited liquid per os, Yes, (%)**	0 (0.0)	1 (4.2)		0 (0)	1 (2.2)	
**Nasopharyngeal swab request, Yes, (%)**	12 (20.7)	7 (29.2)		2 (2.0)	10 (22.2)	<0.001
Positive, Yes, (%)	9 (75.0)	7 (100)		2 (100)	8 (80.0)	
Parainfluenza virus	1 (1.7)	0 (0)		0 (0)	0 (0)	
Rhinovirus	3 (5.2)	2 (8.3)		0 (0)	4 (8.9)	
RSV	5 (8.6)	5 (20.8)		2 (2.0)	4 (8.9)	
**Chest X-ray exams, Yes, (%)**	17 (29.3)	6 (25.0)		5 (5.0)	3 (6.7)	
**White blood cell count exam, Yes, (%)**	40 (69.0)	17 (70.8)		9 (9.0)	9 (20.0)	
WBC/mL, Mean (SD)	11.6 (5.90)	12.7 (6.05)		11.3 (2.94)	10.1 (4.19)	
WBC/mL, Median [Min, Max]	10.9 [3.05, 36.7]	11.5 [5.83, 27.8]		11.8 [7.03, 15.5]	9.63 [5.30, 17.3]	
**C reactive protein exam, Yes, (%)**	46 (79.3)	19 (79.2)		12 (12.0)	10 (22.2)	
mg/L, Mean (SD)	16.4 (19.9)	17.8 (17.6)		11.5 (12.7)	23.8 (20.7)	
mg/L, Median [Min, Max]	8.30 [0.50, 95.0]	12.7 [0.90, 63.4]		8.05 [0.70, 45.0]	26.1 [1.30, 65.5]	
**Procalcitonin, Yes, (%)**	17 (29.3)	17 (70.8)	0.001	3 (3.0)	9 (20.0)	0.001
ug/L, Mean (SD)	0.169 (0.15)	0.645 (1.66)		0.093 (0.042)	0.143 (0.161)	
ug/L, Median [Min, Max]	0.130 [0.050, 0.640]	0.150 [0.040, 7.00]		0.080 [0.060, 0.140]	0.090 [0.040, 0.560]	

^a^ = In three cases no O_2_ saturation request was found; ^b^ = in one case no O_2_ saturation request was found; ^c^ = data missing for five episodes.

**Table 2:** Bronchiolitis treatment for inpatients and outpatients with significant *p* values.

	Inpatients			Outpatients		
	Pre-Intervention Period	Post-Intervention Period	*p*-Value	Pre-Intervention Period	Post-Intervention Period	*p*-Value
**N. of included episodes**	58	24		100	45	
***Primary outcome***						
**Antibiotics, Yes, (%)**	21 (36.2)	3 (12.5)	0.036	1 (1.0)	0 (0)	
**First line therapy, (%)**						
Co-amoxiclav	16 (76.2)	1 (33.3)		0 (0)	0 (0)	
Co-amoxiclav + ceftriaxone	2 (9.5)	1 (33.3)				
Ceftriaxone	2 (9.5)	0 (8.3)		0 (0)	0 (0)	
Azithromycin	1 (4.7)	0 (0)		0 (0)	0 (0)	
Amoxicillin	0 (0)	0 (0)		1 (100)	0 (0)	
Ampicillin + ceftriaxone	0 (0)	1 (33.3)		0 (0)	0 (0)	
***Explorative outcomes***						
**Inhaled glucocorticoids, Yes, (%)**	2 (3.4)	0 (0)		8 (8.0)	0 (0)	
Betamethasone	2 (100)	0 (0)		7 (87.5)	0 (0)	
Budesonide	0 (0)	0 (0)		1 (12.5)	0 (0)	
**Inhaled beta2-agonists, Yes, (%)**	4 (6.9)	5 (20.8)		18 (18.0)	2 (4.4)	0.036
**Inhaled ephineprine, Yes, (%)**	3 (5.2)	0 (0)		0 (0)	0 (0)	

**Figure A1:**
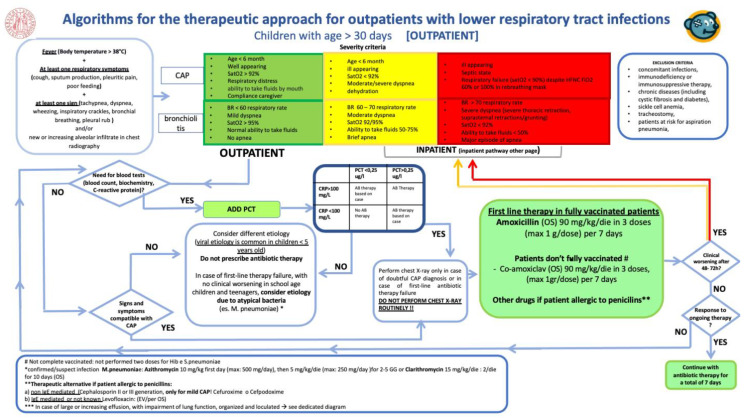
Clinical pathway for lower respiratory tract infection for children older than 30 days of age. Padova, 2019.

## Data Availability

The data used in this study cannot be made available in the manuscript, the supplemental files or in a public repository due to Italian data protection laws. The anonymized datasets generated during and/or analyzed during the current study can be provided on reasonable request, from the corresponding author, after written approval by the Ethical Committee of Padova University Hospital (https://www.aopd.veneto.it/sez,3450, accessed on 14 January 2021).
